# The complete chloroplast genome sequence and phylogenetic analysis of *Juniperus coxii, a* Near Threatened species

**DOI:** 10.1080/23802359.2025.2582527

**Published:** 2025-12-05

**Authors:** Junyao Wu, Chenling Zheng, Fuqin Zhou, Rui Li, Yuan Huang

**Affiliations:** School of Life Sciences, Yunnan Normal University, Kunming, P. R. China

**Keywords:** Complete chloroplast genome, *Juniperus coxii*, phylogenomic analysis

## Abstract

*Juniperus coxii* A. B. Jacks is a near threatened species on the IUCN Red List of Threatened Species. In this study, we reported the complete chloroplast genomes of *J. coxii,* which was 127,588 bp in length, with a GC content of 35.1%. A total of 122 genes were annotated, including 82 protein-coding genes, 36 tRNAs, and 4 rRNAs, and no inverse repeats. Phylogenetic analysis revealed that *J. coxii* and *J. pingii* form a sister group, and were nested in the monophyletic clade of the genus *Juniperus*. The chloroplast genome of *J.coxii* will provide useful genetic resources for further evolutionary studies of the genus *Juniperus.*

## Introduction

*Juniperus coxii* A. B. Jacks, a species in the genus *Juniperus* (Cupressaceae), holds significant economic value as a timber for construction and furniture. It is also used in Tibetan medicine as Xuba (Orhan 2019; Yang et al. 2020). This species is distributed across Tibet and Yunnan (China), Sikkim (India), and northern Myanmar, where it inhabits temperate coniferous forests at altitudes ranging from 2,400 to 3,800 m (Zheng et al. 1987). However, deforestation in China has drastically reduced its distribution by severely degrading its habitat. Consequently, the species is now rarely observed in Yunnan and has been listed as Near Threatened on the IUCN Red List (Li et al. 2013). Thus, urgent conservation efforts are needed to protect *J. coxii* as a valuable germplasm resource.

Historically, *J. coxii* exhibited morphological similarities to *J. recurva* and has been classified as a variety of this species. However, molecular phylogenetic analyses confirmed it as a distinct species with closer evolutionary relationships to other *Juniperus* species (Adams 2000). Molecular identification based on standard DNA barcodes provides an efficient method for species-level differentiation. To date,a complete plastid sequences of multiple *Juniperus* species have been assembled and used to construct the phylogeny of the genus (Miao et al. 2019; Xie et al. 2019; Zhang et al. 2020; Wang and Li 2022). However, the complete chloroplast genome of *J. coxii* has not been reported yet. Assessing genetic diversity through chloroplast genome variations can contribute to both the conservation and enhancement of genetic diversity. In this study, we present the complete chloroplast genome of *J. coxii* and reconstruct phylogenetic trees to elucidate its evolutionary relationships. The results provide a valuable genetic resource for further studies on taxonomy and conservation of *Juniperus*.

## Materials and methods

### Plant material collection and DNA extraction

**Figure 1.**
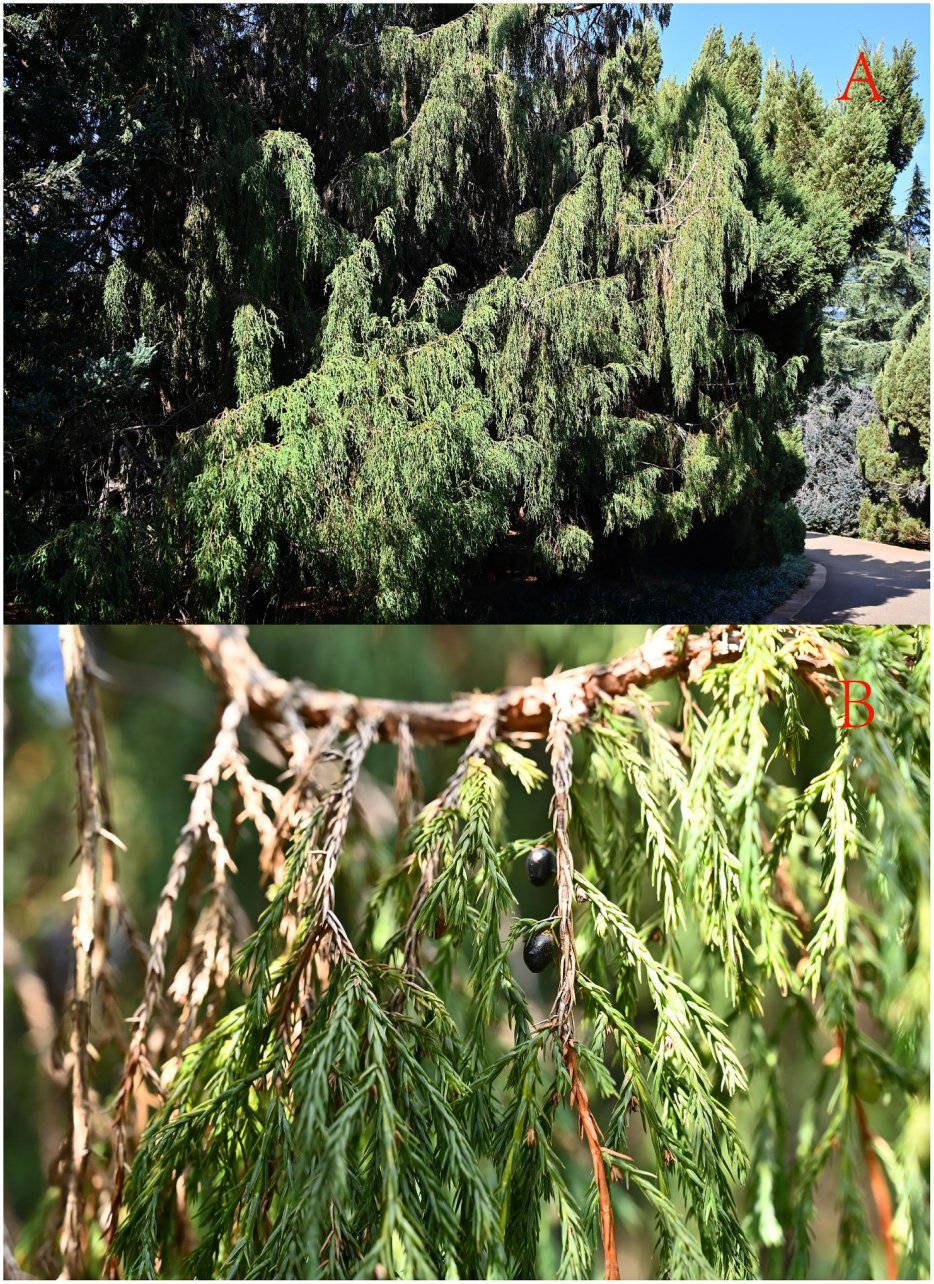
Images of *J. coxii.* Photographed by Yuan Huang in Kunming Botanical Garden (25°07′21′′N, 102°44′29′′E). (a) The whole plant. (b) Leaves are squamiform, straight or slightly incurved. Fruits are egg-shaped, brown in the first year, and ripening to a dark purple the second.

Fresh leaves of *J. coxii* (Figure 1) were collected from the Kunming Botanical Garden (Yunnan, China; coordinates: 25°07′21′′N, 102°44′29′′E). The collection was conducted in accordance with the research policies of the International Union for Conservation of Nature (IUCN) for species at risk of extinction. A voucher specimen of *J. coxii* was identified by Yuan Huang, and deposited in the herbarium of Yunnan Normal University (Kunming, China; Jianlin Hang, hjlynnu@163.com) under voucher number Y-25. Healthy, pest-free mature leaves were picked, rinsed with tap water, dried, and used for DNA extraction *via* a modified CTAB method (Porebski et al. 1997).

### Genome sequencing assembly and annotation

A short-insert library for Illumina paired-end sequencing was prepared. The library was sequenced on an Illumina HiSeq X Ten platform at the Molecular Biology Experimental Platform of the Germplasm Bank of Wild Species. The complete chloroplast genome of *J. coxii* was assembled using NOVOPlasty v2.7.2 (Dierckxsens et al. 2017), with *J. pingii* as a reference genome (GenBank accession No. NC_065034). The assembled chloroplast genome was annotated using Geneious v2020.1.1 (Kearse et al. 2012), and the annotated sequence was submitted to the GenBank database under the accession number (OR086071). The circular map of the *J. coxii* chloroplast genome and the schematic map of the cis and trans-splicing genes were generated using GeSeq (https://chlorobox.mpimp-golm.mpg.de/geseq.html) (Shi et al. 2019).

### Phylogenetic tree construction

To explore the phylogenetic position of *J. coxii* within the genus *Juniperus*, we performed multiple sequence alignments of the complete chloroplast genomes of 24 species from the Cupressaceae using MAFFT v7.47 software (Yang et al. 2012; Katoh and Standley 2013).

We retrieved the chloroplast genomes of 20 representative *Juniperus* species from NCBI GenBank and selected four species from two related genera that are most closely related to *J. coxii* as outgroups for reconstructing phylogenetic trees (2 *Callitropsis* species and 2 *Cupressus* species). The maximum likelihood (ML) tree was constructed using IQ-TREE v1.6.10 (Nguyen et al. 2015), under the TVM + F + R2 best-fit model (Kalyaanamoorthy et al. 2017). Branch support was evaluated using ultrafast bootstrap (UFBoot) (Hoang et al. 2018) and SH-like approximate likelihood ratio test (SH-aLRT) (Guindon et al. 2010) with 10,000 replicates. The phylogenetic tree was visualized using FigTree (https://github.com/rambaut/figtree).

## Results

### Characteristics of chloroplast genome

**Figure 2.**
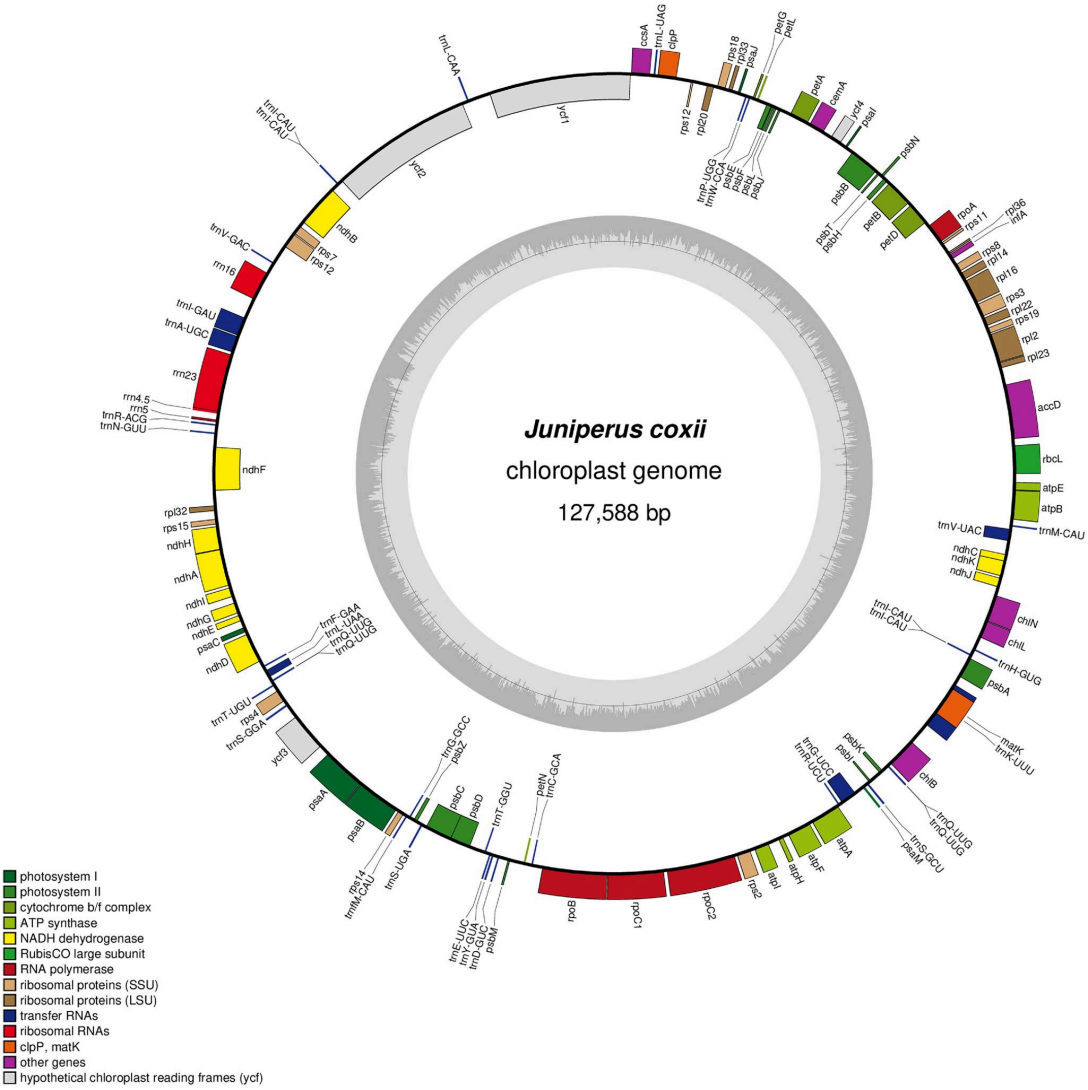
Chloroplast genome schematic map of *J. coxii*. This figure shows the circular map of the *J. coxii* complete chloroplast genome . Differently colored boxes on the outer circle represent genes. The clockwise and counter-clockwise genes transcribed are drawn inside and outside of the circle, respectively. The black color region inside the inner circle indicates the GC content along the genome. In the bottom left corner, the map shows the key for the functional classification of the genes.

We sequenced the whole genome of *J. coxii* using Illumina Hiseq X Ten platform, achieving an average coverage of 283.8 × (Supplementary Figure 1). As a result, the chloroplast genome of *J. coxii* showed a circular structure, with a length of 127,588 bp and a GC content of 35.1%. A total of 122 genes were annotated, including 82 protein-coding genes, 36 tRNA genes, and 4 rRNA genes. Furthermore, the 122 unique genes could be divided into four types according to their functions: photosynthesis, self-replication, other genes, and genes of unknown function (Supplementary Table S1). Consistent with other *Juniperus* species, the chloroplast genome of *J. coxii* lacks inverted repeats (IRs) (Figure 2), consequently lacking the canonical quadripartite architecture characteristic of most terrestrial plant plastomes (Song et al. 2019). A total of one trans-splicing gene and 9 cis-splicing genes (*rpl*2, *rpl*16, *pet*D, *pet*B, *ndh*B, *ndh*A, *ycf*3, *rpo*C1, *atp*F) were identified (Supplementary Figures 2 and 3).

### Phylogenetic relationship

**Figure 3.**
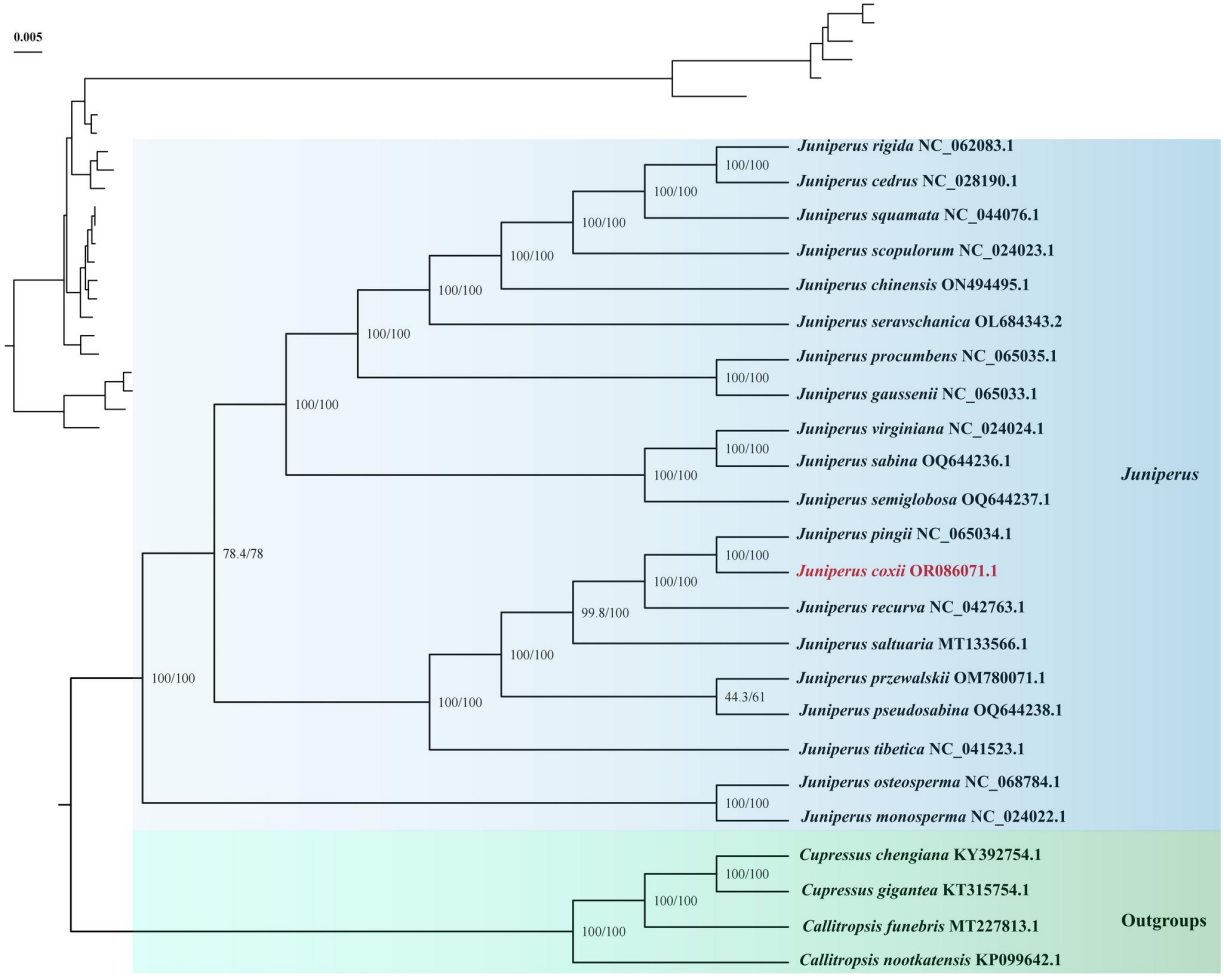
In The ML phylogenetic tree of *J. coxii* and 24 species based on chloroplast genomes. The best-fit model according to the Bayesian information criterion (BIC) is TVM + F + R3. Branch support is tested using ultrafast bootstrap (UFBoot) and SH-like approximate likelihood ratio test (SH-aLRT) with 10,000 replicates. *J. coxii* is marked with an asterisk. The following sequences were used: *J. pingii* NC_065034.1 (Chen et al. 2022), *J. recurva* NC_042763.1 (Song et al. 2019), *J. saltuaria* MT133566.1 (Zhang et al. 2020), *J. przewalskii* OM780071.1, *J. pseudosabina* OQ644238.1, *J. tibetica* NC_041523.1 (Miao et al. 2019), *Cupressus chengiana* KY392754.1, *Cupressus gigantea* KT315754.1 (Li et al. 2016), *Callitropsis funebris* MT227813.1 (Ping et al. 2021), *Callitropsis nootkatensis* KP099642.1, *J. monosperma* NC_024022.1 (Guo et al. 2014), *J. osteosperma* NC_068784.1, *J. sabina* OQ644236.1, *J. virginiana* NC_024024.1 (Guo et al. 2014), *J. semiglobosa* OQ644237.1, *J. gaussenii* NC_065033.1 (Chen et al. 2022), *J. procumbens* NC_065035.1 (Chen et al. 2022), *J. cedrus* NC_028190.1 (Guo et al. 2016), *J. rigida* NC_062083.1 (Wang and Li 2022), *J. squamata* NC_044076.1 (Xie et al. 2019), *J. scopulorum* NC_024023.1, *J. chinensis* ON494495.1 (Chen et al. 2022), *J. seravschanica* OL684343.2 (Yermagambetova et al. 2023).

To reveal the phylogenetic relationship of *J. coxii* with other *Juniperus* species, we performed a phylogenetic analysis based on 20 chloroplast genomes of *Juniperus*, 2 *Cupressus* species, and 2 *Callitropsis* species (*Cupressus chengiana*, *Cupressus gigantea*, *Callitropsis funebris* and *Callitropsis nootkatensis* as outgroups) (Figure 3). The phylogenetic tree showed that the *Juniperus* and four outgroup species each formed a highly supported monophyletic group. The *Juniperus* branch was divided into two clades, with a small clade containing *J. monosperma* and *J. osteosperma*, and a larger clade divided into two clades. Within this large clade , *J. coxii*, *J. pingii*, *J. recurva*, *J. saltuaria*, *J. przewalskii*, *J. pseudosabina* and *J. tibetica* formed a clade. Notably, *J. coxii* was sister to *J. pingii* with a full support value.

## Discussion and conclusion

In this study, the complete chloroplast genome of *J. coxii* was sequenced, assembled, and annotated for the first time. The genome is 127,588 bp in length and contains a total of 122 annotated genes. Similar to other *Juniperus* species, inverted repeat regions were absent from the *J. coxii* complete chloroplast genome (Xie et al. 2019). Interestingly, *J. coxii* was historically classified as a variety of *J. recurva*, but our phylogenetic analysis revealed that *J. coxii* is more closely related to *J. pingii* than to *J. recurva*. This result suggests that *J. coxii* is an independent species rather than a variant of *J. recurva*. The phylogenetic tree supports the classification of *J. monosperma* and *J. osteosperma* as a distinct clade, consistent with a previous comprehensive phylogenetic study of *Juniperus* (Liu et al. 2024). A larger clade includes *J. coxii, J. pingii, J. recurva, J. saltuaria, J. przewalskii, J. pseudosabina and J. tibetica.* This grouping was highly consistent with a previous phylogenetic study on seven *Juniperus* species from Kazakhstan (Almerekova et al. 2024).

The complete chloroplast genome of *J. coxii* provides a valuable genetic resource for further phylogenetic and evolutionary studies in *Juniperus*, and will aid in developing conservation strategies for this ecologically and economically important species.

## Supplementary Material

Supplementary.docx

## Data Availability

The genome sequence data that support the findings of this study are openly available in GenBank of NCBI at https://www.ncbi.nlm.nih.gov/ under accession no. OR086071 (*J. coxii*). The associated BioProject, SRA, and BioSample numbers are PRJNA979966, SRR24825704 and SAMN35617921, respectively.
