# Supplementary material for: The complete chloroplast genome sequence and phylogenetic analysis of Juniperus coxii, a Near Threatened species
Source: Mitochondrial DNA B Resour. 2025 Dec 5;11(1):33–8. doi: 10.1080/23802359.2025.2582527 (PMC12683768; doi:10.1080/23802359.2025.2582527)
Supplement: Supplementary.docx [file TMDN_A_2582527_SM7632.docx]

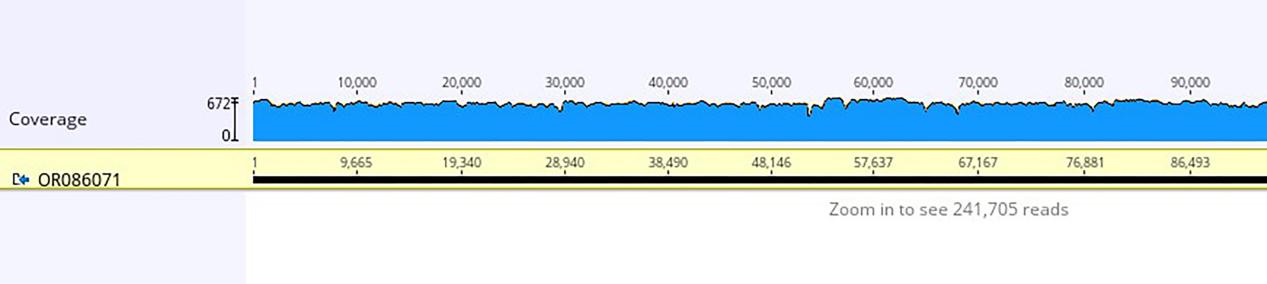


**Figure S1**. The coverage figure of the complete chloroplast genomes of *J.coxii*

**Table S1** Gene annotation of the chloroplast genomes of *J.coxii*

| Category of genes | Group of genes | Name of genes |
| --- | --- | --- |
| Genes for photo-synthesis | Subunits of photosystem I | *psa*A, *psa*B, *psa*C, *psa*I, *psa*J, *psaM* |
|  | Subunits of photosystem II | *psb*A, *psb*B, *psb*C*, psb*D*, psb*E, *psb*F, *psb*H, *psb*I, *psb*J, *psb*K, *psb*L, *psb*M, *psb*N, *psb*T, *psb*Z |
|  | Subunits of NADH-dehydrogenase | *ndh*A*, *ndh*B*, *ndh*C, *ndh*D, *ndh*E, *ndh*F, *ndh*G, *ndh*H, *ndh*I, *ndh*J, *ndh*K |
|  | Subunits of cytochrome b/f complex | *pet*A, *pet*B*, *pet*D*, *pet*G, *pet*L, *pet*N |
|  | Subunits of ATP synthase | *atp*A, *atp*B, *atp*E, *atp*F*, *atp*H, *atp*I |
|  | Large subunit of rubisco | *rbc*L |
|  | Subunits photochlorophyllide reductase | *chl*B, *chl*L, *chl*N |
| Self-replication genes | Proteins of large ribosomal subunit | *rpl*14, *rpl*16*, *rpl*2*, *rpl*20, *rpl*22, *rpl*23*, rpl3*2, *rpl*33, *rpl*36 |
|  | Proteins of small ribosomal subunit | *rps*11, *rps*12**, *rps*14, *rps*15, *rps*18, *rps*19, *rps*2, *rps*3, *rps*4, *rps*7, *rps*8 |
|  | Subunits of RNA polymerase | *rpo*A, *rpo*B, *rpo*C1**, rpo*C2 |
|  | Ribosomal RNAs | *rrn*16, *rrn*23, *rrn*4.5, *rrn*5 |
|  | Transfer RNAs | *trn*A-UGC*, *trn*C-GCA, *trn*D-GUC*, trn*E-UUC, *trn*F-GAA, *trn*G-GCC, *trn*G-UCC**, trn*H-GUG, *trn*I-CAU(4), *trn*I-GAU*, *trn*K-UUU*, *trn*L-CAA, *trn*L-UAA*, *trn*L-UAG, *trn*M-CAU, *trn*N-GUU, *trn*P-UGG, *trn*Q-UUG(4), *trn*R-ACG, *trn*R-UCU, *trn*S-GCU, *trn*S-GGA, *trn*S-UGA, *trn*T-GGU, *trn*T-UGU, *trn*V-GAC, *trnV*-UAC*, *trn*W-CCA, *trn*Y-GUA, *trnf*M-CAU |
| Other genes | Maturase | *mat*K |
|  | Protease | *clp*P |
|  | Envelope membrane protein | *cem*A |
|  | Acetyl-CoA carboxylase | *acc*D |
|  | c-type cytochrome synthesis gene | *ccs*A |
|  | Translation initiation factor | *inf*A |
|  | other | *-* |
| Unknown functional genes | Conserved hypothetical chloroplast ORF | *ycf*1, *ycf*2, *ycf*3**, *ycf*4 |

Notes: Gene*: Gene with one introns; Gene**: Gene with two introns; #Gene: Pseudo gene; Gene(2): Number of copies of multi-copy genes;


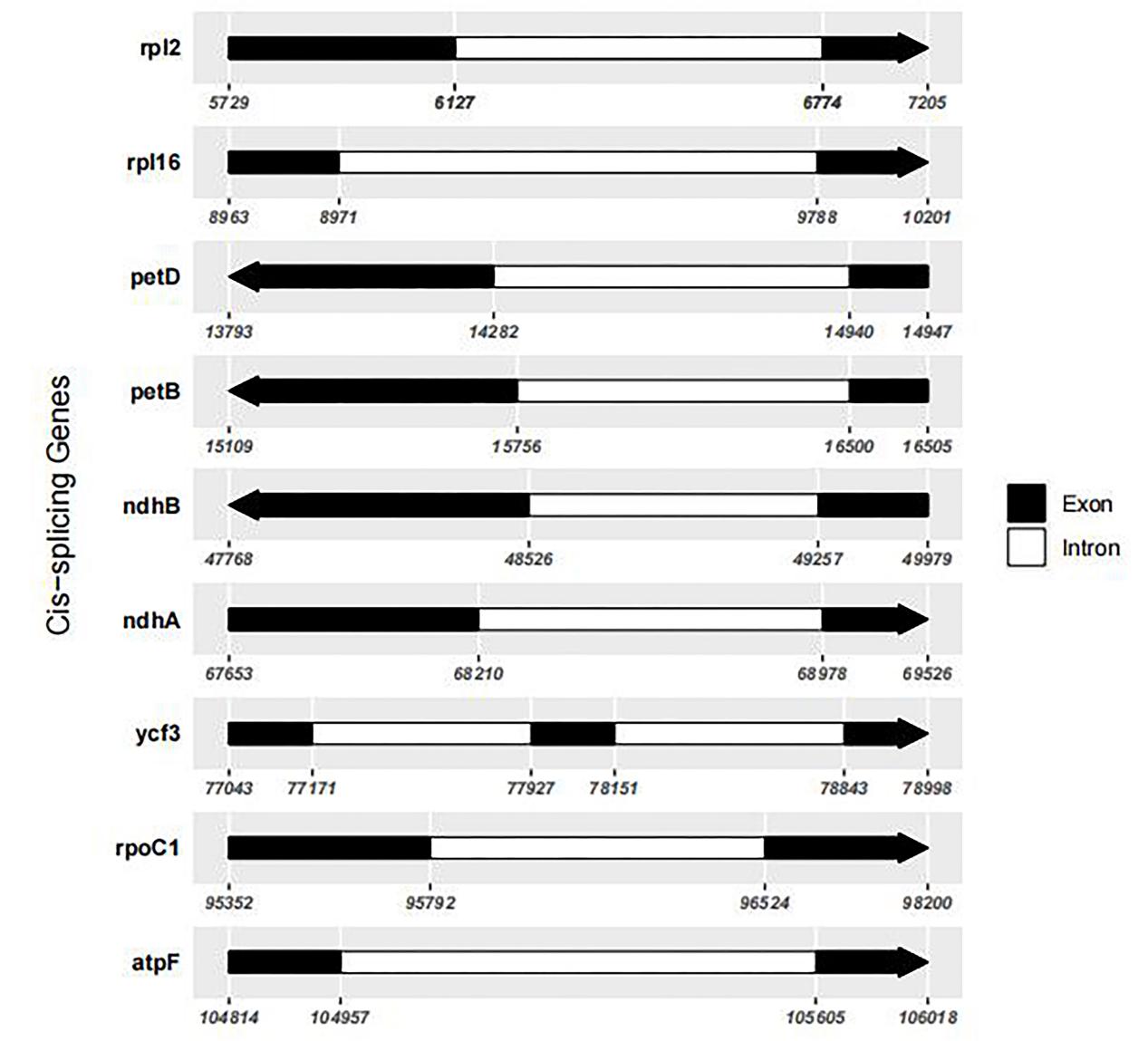


**Figure S2**. Schematic map of the cis-splicing genes in the chloroplast genomes of *J.coxii*


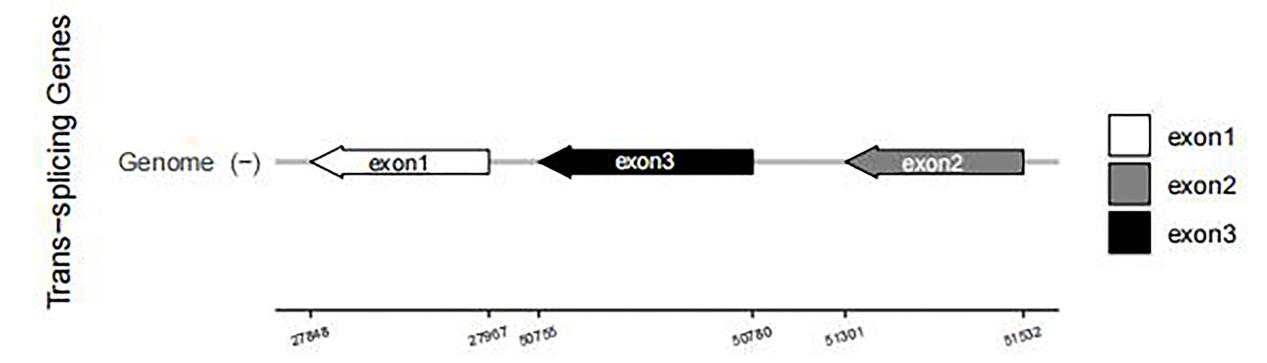


**Figure S3**. Schematic map of the trans-splicing genes in the chloroplast genomes of *J.coxii*
